# Atypical Presentation of Constrictive Pericarditis With Constrictive Physiology on Cardiac MRI

**DOI:** 10.7759/cureus.41626

**Published:** 2023-07-10

**Authors:** Michael Vaysblat, Gurjaipaul Kang, Beatrice Panjwani, Manile Dastagir, Dennis T Roarke

**Affiliations:** 1 Internal Medicine, Northwell Health, New York, USA; 2 Radiology, Northwell Health, New York, USA; 3 Internal Medicine, North Shore University Hospital, New York, USA; 4 Medicine, Northwell Health, New York, USA

**Keywords:** echocardiogram, cardiac magnetic resonance (cmr), pericardiectomy, constrictictive pericarditis, constrictive physiology

## Abstract

Constrictive pericarditis (CP) is a disease primarily affecting the pericardial sac surrounding the heart. The constrictive physiology placed on the heart chambers can lead to clinical presentations mimicking heart failure and possibly primary liver disease. The diagnosis can often be missed and attributed to other etiologies until the patient undergoes extensive workup to rule out each potential etiology. Diagnosis can be delayed, leading to suboptimal outcomes and mortality rates. Here, we present a case of CP initially presenting with bilateral lower extremity and scrotal edema, initially attributed to alcoholic liver cirrhosis given the patient’s history of alcohol abuse. Subsequent abdominal imaging found no evidence of cirrhosis, coupled with grossly normal echocardiogram that led to extensive workup and eventually the diagnosis of CP based on cardiac MRI. The patient later underwent pericardiectomy and made a full recovery. This case highlights the often ambiguous presentation of CP, the utility of cardiac MRI in diagnosis, and the need for specific criteria to help guide future diagnoses as imaging modalities continue to evolve.

## Introduction

Constrictive pericarditis (CP) is a cardiac disease characterized by a thickened pericardium, sometimes with calcifications, typically caused by chronic inflammation [1,2]. While not always present, a thickened pericardium can lead to constrictive physiology on the heart chambers themselves, which can manifest clinically as left- or right-sided heart failure [3]. This can make diagnosis challenging as initial diagnostic testing may miss the characteristics of CP, leading to an extensive workup. Echocardiography can sometimes reveal characteristics suggesting CP and signs of constrictive physiology, but its sensitivity widely varies. Studies have shown that cardiac MRI (CMR) has proven to be much more sensitive in recognizing signs of CP and constrictive physiology. Patients must typically go through extensive workup ruling out alternative diagnoses, such as primary left- or right-sided heart failure, pulmonary hypertension, or primary liver cirrhosis prior to arriving at the correct conclusion. This can lead to a delayed time to diagnosis, which can complicate outcomes as the literature has shown that patients may have better mortality rates when pericardiectomy is performed sooner [1]. Here, we present a case of CP that presented with chronic lower extremity and scrotal edema, which was initially attributed to presumed alcoholic liver cirrhosis. After extensive workup, CMR showed CP with constrictive physiology, and the patient later underwent a liver biopsy confirming congestive hepatopathy. After diagnostic confirmation, he was treated with surgical pericardiectomy and made a full recovery. This case highlights the importance of maintaining a broad list of differential diagnoses when approaching even common clinical presentations and using a systematic approach to arrive at the correct conclusion.

## Case presentation

**Figure 1:**
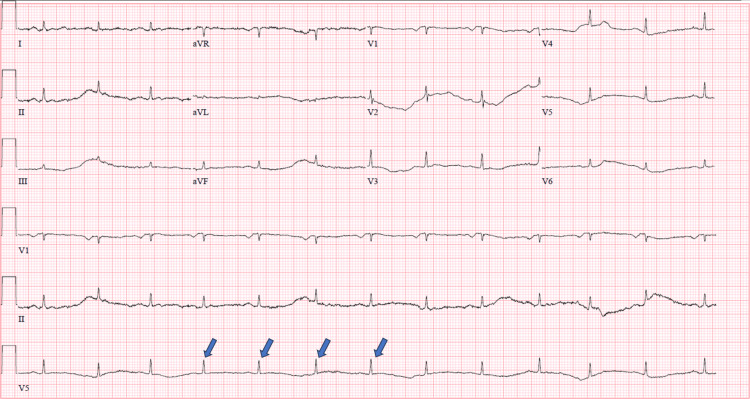
Electrocardiogram showing low-voltage QRS complexes (several shown with blue arrows)

A 59-year-old man with a history of alcoholic liver cirrhosis, pancreatitis, type 2 diabetes mellitus, chronic obstructive pulmonary disease (GOLD Stage I), hypothyroidism, and chronic bilateral loculated pleural effusions presented to the hospital with bilateral lower extremity and scrotal edema for the past month. He reported a past history of alcohol use, usually having two to three drinks daily for many years up until quitting two years ago. He denied chest pain, heart palpitations, orthopnea, oliguria, and changes in urination habits. Vital signs on presentation were as follows: temperature 98.2 °F, heart rate 86 beats per minute, blood pressure 108/72 mmHg, respiration rate 16 breaths per minute, and oxygen saturation 99% on room air. Physical exam was remarkable for clear lungs on auscultation bilaterally, normal heart sounds, and 3+ bilateral lower extremity pitting edema up to the patient’s thighs and scrotum. EKG was significant for low-voltage QRS complexes (Figure 1). Initial laboratory studies, including complete blood count, complete metabolic panel, thyroid-stimulating hormone, and pro-brain natriuretic peptide, were within normal limits. Chest X-ray was unremarkable with no signs of pneumothorax or pulmonary edema. The patient was taking oral furosemide at home for chronic lower extremity swelling and was recently taken off spironolactone after hospitalization for hyperkalemia several months prior. He was started on intravenous (IV) furosemide for diuresis. Ultrasonography of the scrotum revealed scrotal wall thickening suggestive of edema, while bilateral lower extremity duplex was negative for deep vein thrombosis. Ultrasonography of the abdomen was remarkable for hepatic steatosis, pancreatic ductal dilatation with intraluminal calculi, dilated inferior vena cava (IVC) and hepatic veins, and absence of ascites. Hepatology was consulted and recommended starting metolazone for better urine output and midodrine to support his soft blood pressures for continued diuresis. His transthoracic echocardiogram (TTE) showed normal left and right ventricular systolic function with a left ventricular ejection fraction (LVEF) of 55-60%, but right ventricular systolic pressure (RVSP) and pulmonary pressures were unable to be estimated due to insufficient tricuspid regurgitation. Anti-nuclear antibody, anti-cyclic citrullinated peptide, anti-double-stranded DNA, rheumatoid factor, anti-centromere, cytoplasmic and perinuclear anti-neutrophil cytoplasmic antibodies, anti-Sjogren's syndrome A (SSA)/Sjogren's syndrome B (SSB), and QuantiFERON-TB Gold testing all came back negative.

**Figure 2:**
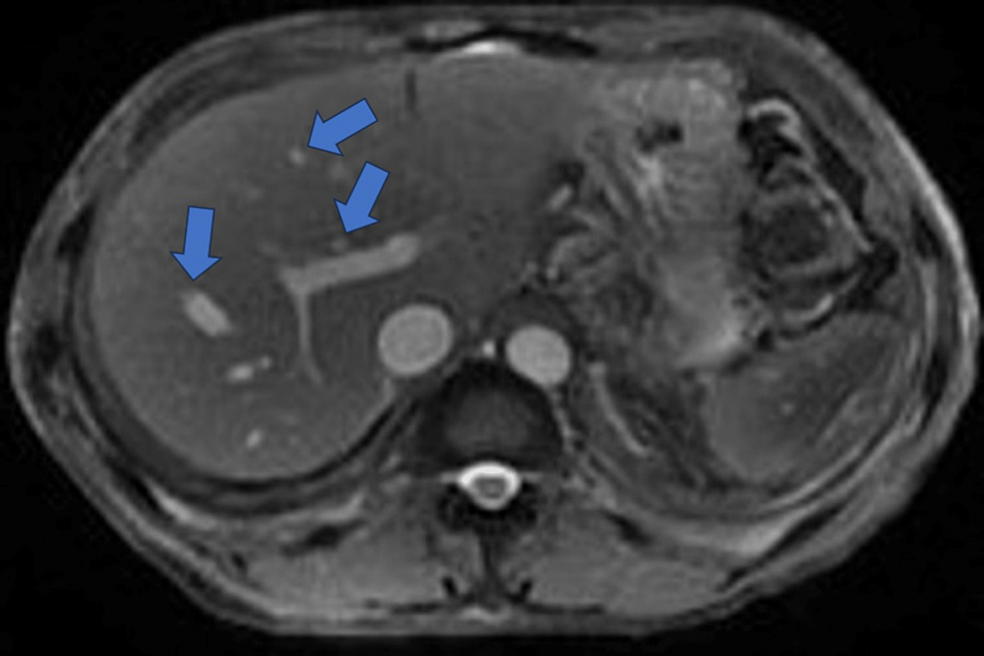
MRI abdomen with and without IV contrast showing hepatic steatosis (blue arrows)

Hepatology recommended MRI abdomen with and without IV contrast, which revealed hepatic steatosis with no enhancing liver lesions or cirrhosis, large colonic stool burden, trace loculated ascites, sequela of chronic pancreatitis including parenchymal atrophy, and trace bilateral loculated pleural effusions (Figure 2). After several days of diuresis with adequate urine output, the patient’s edema only had minimal improvement even after transition to IV bumetanide. The patient also developed rising creatinine levels from 0.79 mg/dL up to 1.69 mg/dL, for which nephrology was consulted. His rising creatinine was attributed to prerenal acute kidney injury most likely secondary to episodic hypotension in the setting of diuresis. Bumetanide and metolazone were temporarily discontinued with subsequent improvement of serum creatinine levels to 0.87 mg/dL after several days.

**Figure 3:**
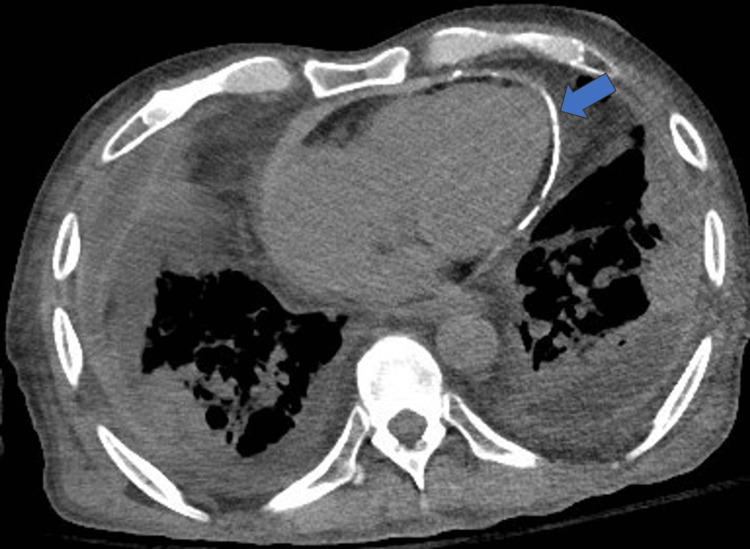
CT chest without IV contrast showing pericardial calcifications (blue arrow)

Given the previously noted findings of dilated IVC and hepatic veins on the abdominal ultrasound, there was a concern for pelvic vein thrombosis or compression of the lower body vasculature or lymphatics as potential etiologies of the patient’s edema. An MRI venogram of the pelvis with and without contrast showed no evidence of thrombosis or compression. The patient began to develop worsening shortness of breath with desaturation to 87%. He promptly received IV bumetanide and was placed on nasal cannula at 3 L with immediate improvement. CT chest without contrast was urgently performed and showed diffuse pericardial thickening and calcification, suggesting chronic pericarditis and an enlarged pulmonary artery concerning for pulmonary hypertension (Figure 3). At this point, the primary etiology of the patient’s swelling was thought to be more likely secondary to pulmonary hypertension rather than primary liver cirrhosis. Pulmonology recommended a ventilation-perfusion scan to rule out chronic thromboembolic pulmonary hypertension, which was negative, and intravenous bumetanide was reinitiated. 

Repeat TTE could not estimate RVSP, so cardiology was consulted for right heart catheterization, which measured a pulmonary arterial pressure of 35/23 mmHg and mean pulmonary pressure of 25 mmHg, confirming the presence of pulmonary hypertension. Cardiology recommended cardiac MRI to better characterize the patient’s cardiac structures and anatomy, which confirmed the presence of chronic pericarditis with constriction, specifically showing paradoxical movement of the interventricular septum (Video 1). Cardiothoracic surgery was consulted for pericardiectomy, which was performed with milrinone support and had no complications. As a part of preoperative workup, the patient underwent liver biopsy, with pathology later showing marked zone 3 dilation of the sinusoids and portal, perisinusoidal, and central zone fibrosis. These findings suggest congestive hepatopathy with no cirrhosis. The patient was later discharged with close outpatient follow-up with cardiology, cardiothoracic surgery, and hepatology.

**Video 1:** Cardiac MRI Cine Video showing paradoxical movement of the interventricular septum

## Discussion

CP can have variable presentations due to its vague symptmkatology and can lead to various complications, including decreased cardiac output and eventual congestive heart failure. Diagnosing CP can be challenging as presentations can mimic many other conditions that can cause heart failure or primary liver disease. In this case presentation, the patient was thought to have liver cirrhosis secondary to his history of chronic alcohol use, which led to his chronic leg edema. His initial presentation with scrotal and bilateral lower extremity edema was initially attributed to a primary liver etiology. Acute decompensation of his liver cirrhosis was considered, but the patient did not have physical exam findings, such as jaundice, or any lab abnormalities, such as elevated liver function tests or bilirubin, supporting this diagnosis. MRI of the abdomen performed later in the hospital course showed hepatic steatosis but did not show any signs of liver cirrhosis, making a cardiac etiology more likely. Initial TTE was unremarkable, but pulmonary arterial pressures were unable to be assessed, making pulmonary hypertension difficult to rule out until the patient underwent right heart catheterization for definitive diagnosis. The ambiguous presenting symptoms typically lead to an extensive workup before determining the definitive etiology.

According to the literature, CP is often diagnosed late, with a mean time of symptom onset to diagnosis of 24 months, with later diagnoses associated with poorer outcomes [1]. CP can present with anasarca, heart failure, ascites, and many nonspecific symptoms, such as nausea, dyspepsia, liver disease, and weight loss. Etiologies include viral infection, tuberculosis, end-stage renal disease, malignancy, trauma, history of pericardiectomy, and rheumatological disease [2]. This patient was found to have none of these potential etiologies and most likely had idiopathic disease, which is most commonly seen [4]. While clinical diagnosis may be difficult, imaging can help rule out common etiologies. A 2014 study found that transthoracic echocardiography can diagnose CP with sensitivities ranging from 64% to 93% [4]. Welch et al. investigated which findings seen on echocardiography were most associated with CP. Among patients with surgically diagnosed CP, five echocardiographic features were studied, with independent associations found in three: ventricular septal shift, elevated medial mitral valve e’ velocities, and elevated hepatic vein expiratory diastolic reversal ratios. Patients with CP tend to have higher LVEF than patients with non-constrictive pericarditis, with higher rates of pericardial thickening, change in the ventricular contour, and tethering of the right ventricular free wall. Higher e’ velocities at the medial mitral annulus were noted in CP patients as well, typically because left ventricular diastolic pressure tends to be elevated in CP, and most left ventricular filling occurs during early diastole [4,5]. CP patients also tend to have higher systolic forward velocities in the hepatic veins but lower diastolic forward velocities [2]. These variables can serve as a foundation for the establishment of formal criteria or guidelines that can be used to diagnose CP, especially as sensitivities tend to vary significantly with TTE. While some variability may be operator dependent or attributed to the presence of adequate cardiac windows, close attention to these variables may lead to improved sensitivities for CP with TTE. A study by Yang et al. investigated the utility of the ratio between the right atrial pressure (RAP) and pulmonary artery wedge pressure (PAWP), finding that this can estimate the degree of pericardial restraint versus restrictive pericardium [6]. The RAP/PCWP ratio was found to be associated with long-term survival in patients that received pericardiectomy and may serve as a prognostic indicator. 

Cardiac MRI can be somewhat helpful in diagnosis with increased pericardial thickness seen as a common finding, which can also be noted on CT. However, normal pericardial thickness measurements do not necessarily rule out the presence of CP [6,7]. This patient had paradoxical motion of the interventricular septum, which is a common feature of CP seen on imaging. This finding can be seen on echocardiography, although cardiac MRI tends to be more sensitive [8]. Invasive diagnostics, such as catheterization, may help inform diagnosis and should be considered on a case-by-case basis if noninvasive imaging does not yield a concrete diagnosis. With a wide range of noninvasive cardiac imaging techniques available and rapidly developing, there is much data available to clinicians to help identify cases of CP. Future developments in machine learning and refined imaging guidelines for CP may decrease the time to diagnosis and improve outcomes. While CP can be elusive as a diagnosis, maintaining a wide list of differential diagnoses and utilizing imaging techniques effectively will lead to a faster time to diagnosis and better outcomes.

## Conclusions

CP can be a challenging diagnosis due to its symptomatology overlapping with other common presentations. In this case, a patient with many comorbidities and a history of alcohol use was found to have CP rather than a primary liver disease as the main etiology of his anasarca and lower extremity edema. This case highlights the necessity of always maintaining a broad differential even when approaching common presentations that may even be supported by clinical history.
